# The alpha 7 nicotinic receptor agonist PHA-543613 hydrochloride inhibits *Porphyromonas gingivalis*-induced expression of interleukin-8 by oral keratinocytes

**DOI:** 10.1007/s00011-014-0725-5

**Published:** 2014-03-08

**Authors:** Alexandrea Macpherson, Noha Zoheir, Raja Azman Awang, Shauna Culshaw, Gordon Ramage, David F. Lappin, Christopher J. Nile

**Affiliations:** Infection and Immunity Research Group, Glasgow Dental School, School of Medicine, College of Medical, Veterinary and Life Sciences, University of Glasgow, Level 9, 378 Sauchiehall Street, Glasgow, G2 3JZ UK

**Keywords:** Oral keratinocytes, Alpha 7 nicotinic receptor, Acetylcholine, Periodontal disease, Inflammation

## Abstract

**Objective:**

The alpha 7 nicotinic receptor (α7nAChR) is expressed by oral keratinocytes. α7nAChR activation mediates anti-inflammatory responses. The objective of this study was to determine if α7nAChR activation inhibited pathogen-induced interleukin-8 (IL-8) expression by oral keratinocytes.

**Materials and methods:**

Periodontal tissue expression of α7nAChR was determined by real-time PCR. OKF6/TERT-2 oral keratinocytes were exposed to *Porphyromonas gingivalis* in the presence and absence of a α7nAChR agonist (PHA-543613 hydrochloride) alone or after pre-exposure to a specific α7nAChR antagonist (α-bungarotoxin). Interleukin-8 (IL-8) expression was measured by ELISA and real-time PCR. Phosphorylation of the NF-κB p65 subunit was determined using an NF-κB p65 profiler assay and STAT-3 activation by STAT-3 in-cell ELISA. The release of ACh from oral keratinocytes in response to *P. gingivalis* lipopolysaccharide was determined using a GeneBLAzer M3 CHO-K1-*bla* cell reporter assay.

**Results:**

Expression of α7nAChR mRNA was elevated in diseased periodontal tissue. PHA-543613 hydrochloride inhibited *P. gingivalis*-induced expression of IL-8 at the transcriptional level. This effect was abolished when cells were pre-exposed to a specific α7nAChR antagonist, α-bungarotoxin. PHA-543613 hydrochloride downregulated NF-κB signalling through reduced phosphorylation of the NF-κB p65-subunit. In addition, PHA-543613 hydrochloride promoted STAT-3 signalling by maintenance of phosphorylation. Furthermore, oral keratinocytes upregulated ACh release in response to *P. gingivalis* lipopolysaccharide.

**Conclusion:**

These data suggest that α7nAChR plays a role in regulating the innate immune responses of oral keratinocytes.

## Introduction

The alpha 7 nicotinic acetylcholine receptor (α7nAChR) plays a key role in regulating the ‘cholinergic anti-inflammatory pathway’ [1–3]. The ‘cholinergic anti-inflammatory pathway’ is a mechanism by which the autonomous nervous system can modulate inflammation. The afferent branch of the vagus nerve relays inflammatory signals emanating from tissues to the brain. In response, acetylcholine (ACh) is released via increased efferent vagal nerve activity. The released ACh then binds to α7AChRs on proximal immunocompetent cells and downregulates localised inflammation [4].

Studies using vagotomised animals have shown the importance of the ‘cholinergic anti-inflammatory pathway’ in regulating inflammatory responses to pathogens. In a polymicrobial model of abdominal sepsis, vagotomised animals showed elevated serum levels of tumour necrosis factor alpha (TNF-α) and interleukin (IL)-6 [5]. Similarly, in a mouse model of bacterial lipopolysaccharide (LPS)-induced endotoxic shock, vagotomised animals demonstrated increased levels of both serum and hepatic TNF-α [4]. These studies highlight the functional relevance of the vagus nerve—immune system cross-talk. However, whether the apparent anti-inflammatory effects are exclusively mediated via vagus nerve-derived ACh remains unresolved [6–8]. Indeed, ex vivo, the anti-inflammatory responses of cells to cholinergic agonists are mediated by mechanisms independent of vagus nerve activity [9]. Thus, non-neuronal ACh may also play a role in regulating localised inflammation at sites far removed from the influence of the vagus nerve.

Studies using α7nAChR-deficient (*α7nAChR* ^−*/*−^) animals have shown the key role that α7nAChR plays in regulating the ‘cholinergic anti-inflammatory pathway’. *α7nAChR* ^−/−^ mice intratracheally instilled with 5 mg/ml *Escherichia coli* LPS, or instilled with 10^7^ colony-forming units (CFU) directly into the lung air spaces, demonstrated higher mortality from sepsis-induced lung injury and had more severe lung damage than control *α7nAChR* ^+*/*+^ animals [10]. In addition, *α7nAChR* ^−/−^ mice injected intraperitoneally with LPS were found to be hypersensitive and presented with increased expression levels of TNF-α in serum, liver and the spleen as well as increased levels of IL-1β and IL-6 in serum [11].

α7nAChR is widely expressed by immunocompetent cells which can also synthesise and release ACh [12]. This therefore suggests that both autocrine and paracrine mechanisms are in place to regulate inflammation. In vitro, the pharmacological α7nAChR agonist 3-(2,4-dimethoxybenzylidene) anabaseine (GTS-21) inhibited Toll-like receptor (TLR)-mediated expression of TNF-α and IL-6 from macrophages [13]. Furthermore, activation of α7nAChR has been shown to reduce IL-8 expression by epithelial cells of patients with cystic fibrosis [14] and human colon epithelial cells [15]. In addition, α7nAChR activation reduced TNF-α expression by HBE16 airway epithelial cells [16] and reduced expression of IL-6 and IL-8 from fibroblast-like synoviocytes [17].

The mechanisms by which α7nAChR mediates anti-inflammatory responses have been elucidated in monocytes/macrophages. α7nAChR activation inhibited the transcriptional activity of nuclear factor kappa-light-chain-enhancer of activated B cells (NF-κB) [18]. In addition, α7nAChR activation also stimulated the Janus kinase 2/signal transducer and activator of transcription 3 (JAK-2/STAT-3) pathway, leading to inhibited pro-inflammatory cytokine expression, and promoting IL-10 expression [19]. Therefore, α7nAChR activation can specifically induce downregulated expression of pro-inflammatory cytokines whilst promoting expression of anti-inflammatory cytokines.

Periodontal disease is a chronic inflammatory condition affecting the complex anatomical structures which support the teeth. Although the primary initiating factor is the pathogenic microflora of subgingival plaque, periodontal disease results from an inappropriately excessive and dysregulated immune response to these bacteria. α7nAChR is widely expressed by resident and migratory cells found within the periodontium including oral keratinocytes, fibroblasts, macrophages, neutrophils, B cells and T cells [12, 20–24]. In addition, oral epithelial cells have been demonstrated to express all the components required to synthesise, release and respond to ACh. Gingival epithelial cells have been shown to express the ACh synthesising enzyme, choline acetyltransferase (ChAT) [21], and free ACh has been detected in oral keratinocyte homogenates, culture supernatants [25] and gingival tissue samples [26]. Furthermore, there is tentative evidence to suggest that levels of ACh are up-regulated in inflamed gingival tissue [26]. These observations provide evidence for a non-neuronal cholinergic mechanism operating within periodontal tissues. However, the cellular mechanisms which control the synthesis storage and release of ACh within periodontal tissues remain unknown.

As the host response plays a prominent role in periodontal disease pathogenesis, host response modulation has emerged as a valid treatment concept [27]. Therefore, α7nAChR may be a potential therapeutic target. Previous studies in monocytes have shown that α7nAChR can inhibit *Porphyromonas* *gingivalis-*induced expression of pro-inflammatory cytokines, while promoting expression of anti-inflammatory IL-10 [28]; this suggests that cholinergic mechanisms operate within the periodontium to regulate the immune responses initiated by periodontal pathogens. However, studies in oral keratinocytes have shown that nicotine, acting via nAChRs, can in fact enhance IL-1β and *P. gingivalis* LPS-induced IL-8 release [29]. However, the relative contributions of specific nAChR subunits (such as α7nAChR) were not investigated in detail in this study.

In this study we begin to investigate the role that α7nAChR specifically plays in modulating early immune responses of oral keratinocytes against periodontal pathogens. Oral keratinocytes reside at the gingival sulcus and respond to bacterial challenge via TLRs. Their activation engages the transcription factor NF-κB to increase expression and secretion of cytokines and chemokines, in particular IL-8 (CXCL8). IL-8 plays a pivotal role in the recruitment of immune cells to sites of infection in the periodontium. If this process is not regulated appropriately, it acts as one of the earliest steps in the pathogenesis of periodontal disease. The data show for the first time that specific activation of α7nAChR can modulate the activity of both the NF-κB and STAT-3 transcription factors and negatively regulate *P. gingivalis-*induced IL-8 expression by oral keratinocytes. In addition, the release of ACh from oral keratinocytes is mediated by *P. gingivalis* LPS. These findings suggest that complex cholinergic mechanisms operate within the periodontium. Furthermore, as the oral mucosa shows structural similarities to mucosal tissues in the gut, lungs and many other organ systems, it is interesting to speculate that non-neuronal cholinergic mechanisms may be involved in regulating pathogen-induced inflammation at other mucosal surfaces.

## Materials and methods

### RNA isolation from periodontal tissue

Diseased tissues were obtained from patients with periodontal disease undergoing surgery in the Unit of Periodontics at Glasgow Dental Hospital. Healthy tissues were taken from patients undergoing non-periodontal disease-related mucogingival surgery. Ethical review and approval was provided by the West of Scotland Research Ethics Committee. Written consent was obtained from all participants. The tissue samples were immediately submerged in RNAlater™ (Qiagen, UK) and stored at −80 °C. RNA extraction and purification from tissue samples was carried out using the RNeasy^®^ Fibrous Tissue Kit (Qiagen).

### TaqMan^®^ real-time PCR

mRNA was reverse transcribed to cDNA using the High Capacity RNA-to-cDNA Master Mix (Applied Biosystems, UK) according to the manufacturer’s instructions. Real-time PCR was carried out using TaqMan^®^ Gene Expression Master Mix (Applied Biosystems) and TaqMan^®^ Gene Expression Assay Mix for the following genes—*α7nAChR* = Hs01063373_m1 and *POLR2A* (RNA polymerase II) = Hs00172187_m1 (both Life Technologies, UK). Analysis of samples was performed in duplicate in a 96-well plate format on a MX3000P™ real-time PCR machine (Stratagene, UK). Data were analysed using MxPro-Mx3000P software, version 4.10 (Stratagene). Relative expression of α7nAChR mRNA was calculated using the 2^−ΔCT^ method [30].

### *P. gingivalis* culture

*P. gingivalis* biofilms were prepared as follows. *P. gingivalis* ATCC 33277 was cultured at 37 °C in Schaedler anaerobe broth (Oxoid, UK) for 2 days. The bacteria were then washed by centrifugation in PBS and standardised to 1 × 10^7^ cells/ml in artificial saliva [31]. *P. gingivalis* (500 µl) suspension was then cultured on Thermanox^®^ plastic coverslips (NUNC, UK) at 37 °C in an anaerobic environment for 4 days. Artificial saliva was replaced daily.

Heat-killed *P. gingivalis* was obtained by incubating a 1 × 10^8^ CFU/ml suspension of bacteria in PBS at 56 °C for 30 min.

### OKF6/TERT-2 cell culture

OKF6/TERT-2 oral keratinocytes were a gift from the Rheinwald Laboratory (Brigham and Women’s Hospital, Boston, USA). OKF6/TERT-2 cells were cultured in keratinocyte serum-free medium (KSFM) supplemented with 25 μg/ml bovine pituitary extract, 0.2 ng/ml epidermal growth factor (Life Technologies), 2 mM l-glutamine, 100 IU/ml penicillin, 100 mg/ml streptomycin and 0.4 μM calcium chloride (Sigma-Aldrich, UK) at 37 °C with 5 % CO_2_. The growth media were changed at three-day intervals and cells were passaged when they reached 70–80 % confluence.

For in vitro experiments, 1 × 10^5^ OKF6/TERT-2 cells were seeded into 24-well plates. The plates were then incubated at 37 °C with 5 % CO_2_ overnight. The following day, the media in each well were changed to defined keratinocyte serum-free media (d-KSFM) (Invitrogen, UK). To stimulate the cells, the Thermanox^®^ coverslips, with the live *P. gingivalis* biofilms, were attached to Millicell^®^ cell culture inserts (Millipore, UK) using Vaseline^®^ and suspended above the cell monolayer. This was conducted in the presence of varying pharmacological concentrations (10, 100, 1,000 nM) of a commercially available α7nAChR agonist, PHA-543613 hydrochloride (HCl; Tocris, UK). For control purposes, cells were left unstimulated or exposed to 1,000 nM PHA-543613 HCl in the absence of biofilm. To confirm the specific role of α7nAChR, experiments were repeated in which 10 nM of α7nAChR antagonist, α-bungarotoxin (Tocris), was added to cells 30 min prior to PHA-543613 HCl. The plates were then incubated at 37 °C with 5 % CO_2_ for 4 or 24 h. At each time point, the bathing supernatant was harvested for analysis by ELISA and the cell monolayer lysed with RLT buffer (Qiagen) for RNA isolation.

### SYBR^®^ green real-time PCR

To measure changes in IL-8 mRNA expression, mRNA was extracted from cell monolayers using an RNeasy^®^ mini kit (Qiagen). RNA was reverse transcribed to cDNA as described previously. IL-8 mRNA was then measured using SYBR^®^ green real-time PCR technology with the following primers—GAPDH: Forward: CAAGGCTGAGAACGGAAG and Reverse: GGTGGTGAAGACGCCAGT; IL-8: Forward: CAGAGACAGCAGAGCACACAA and Reverse: TTAGCACTCCTTGGCAAAAC. Real-time PCR was performed on a MX3000P™ real-time PCR machine (Stratagene). Data were analysed using MxPro-MX3000P software, version 4.10 (Stratagene). Relative expression of IL-8 mRNA was calculated using the 2^−ΔΔCT^ method for graphical purposes; however, the 2^−ΔCT^ values were used for statistical analysis.

### IL-8 ELISA

Measurement of IL-8 release into bathing supernatants was performed using the human IL-8 Cytoset (Invitrogen) according to the manufacturer’s recommendations. Briefly, monoclonal antibodies specific for IL-8 were pre-coated onto a MaxiSorp F96 Immunoplate (NUNC, Denmark) and incubated overnight at 4 °C. The following day, the plate was washed with PBS-0.05 % Tween 20 and 100 μl of standards and samples added to each well along with 50 μl of working detection antibody. The plate was incubated for 2 h at room temperature with continual shaking and then washed 5 times before the addition of 100 μl of streptavidin-HRP. The plate was incubated for a further 30 min, washed and the reaction developed with 100 μl of TMB (3,3′,5,5′-tetra-methylbenzidine) (KPL, USA). The intensity of the colour was read at both 650 and 450 nm using a Fluostar Omega^®^ microplate reader (BMG Labtech, Germany). Data analysis was performed using BMG Analysis Software (BMG Labtech).

### GeneBLAzer M3 CHO-K1-*bla* cell reporter assay

To measure ACh release, 1 × 10^5^ OKF6/TERT-2 cells were seeded into 24-well plates and incubated at 37 °C with 5 % CO_2_ overnight. The following day, the media was replaced with media containing PHA-543613 HCl (10, 100 and 1,000 nM) or *P. gingivalis* LPS (0.1 and 1 μg/ml) and the cells were incubated for 4 h. Media were then changed to media containing 0.5 mM physostigmine hemisulphate (an ACh esterase inhibitor) and incubated for a further 10 min before harvesting the bathing supernatants. ACh release into supernatants was determined using a GeneBLAzer M3 CHO-K1-*bla* cell reporter assay (Invitrogen) as described previously [32]. The experiments were performed in duplicate on three separate occasions. The average response for each experiment was then determined along with the mean of all three experiments. These values were then used to standardise the data set. The data presented show the total ACh (nM) released during a 10-min period.

### Fast-activated cell-based ELISA (FACE™) NF-κB p65 profiler

The effect of PHA-543613 HCl on the activation of NF-κB was investigated using the FACE™ NF-κB p65 profiler (Active Motif, UK). OKF6/TERT-2 cells were seeded into 96-well plates at a density of 1 × 10^5^ cells/well and left to adhere overnight at 37 °C with 5 % CO_2_. The following day, cells were stimulated for 30 min with either 1,000 nM PHA-543613 HCl alone, dead (heat-killed) *P. gingivalis* alone (MOI = 200) or in the presence of varying concentrations (10, 100, 1,000 nM) of PHA-543613 HCl. Supernatants were then discarded and the cells fixed with 4 % formaldehyde. Quenching was then performed using 1 % H_2_O_2_ (v/v) and 0.1 % azide (v/v) in PBS. After quenching, cells were incubated with antibody-blocking buffer prior to the addition of 40 μl of primary antibody—anti-phospho-NFκB p65 (S468), anti-phospho-NFκB p65 (S536) or anti-total NFκB p65. For negative control wells, antibody dilution buffer only was added. Cells were then incubated overnight at 4 °C. The following day, the cells were washed and incubated with HRP-conjugated secondary antibody and then exposed to developing solution for 15 min. The absorbance of each well was then measured at 570 nm with a reference wavelength of 655 nm using a Fluostar Omega^®^ microplate reader (BMG Labtech). To correct the data for cell numbers, the cells were washed and a crystal violet assay performed. All data analysis was performed using BMG Analysis Software (BMG Labtech). The experiment was performed in duplicate on three separate occasions. The percentage of the total NF-κB p65 subunit which was phosphorylated at serine 468 or serine 536 was then calculated in accordance with the manufacturer’s instructions. The percentage data were subjected to angular transformation to parametric data for graphical representation and statistical analysis.

To confirm that changes in NF-κB p65 subunit phosphorylation state corresponded with modulation of IL-8 expression, simultaneous experiments were performed in which replicate cultures in 96-well plates were exposed to the same conditions as described above. The supernatants were harvested after 4 h and IL-8 release investigated by ELISA.

### STAT-3 in-cell ELISA

The effect of PHA-543613 HCl on the activation of STAT-3 signalling was investigated using the STAT-3 in-cell ELISA kit (Thermo Scientific, UK). OKF6/TERT-2 cells were seeded into 96-well plates at a density of 1 × 10^4^ cells/well and left to adhere overnight at 37 °C with 5 % CO_2_. The following day, cells were stimulated for 30 min with either 1,000 nM PHA-543613 HCl alone, dead *P. gingivalis* alone (MOI = 200) or dead *P. gingivalis* in the presence of varying concentrations (10, 100 and 1,000 nM) of PHA-543613 HCl. Supernatants were then discarded and the cells fixed with 4 % formaldehyde. The cells were then permeabilised and quenching performed with 1 % H_2_O_2_ (v/v) and 0.1 % azide (v/v). After quenching, cells were incubated with antibody-blocking buffer prior to the addition of 50 μl of anti-phospho-STAT3 (Y705) or anti-total STAT3 primary antibody. For negative control wells, antibody dilution buffer only was added. Cells were then incubated overnight at 4 °C. The following day, the cells were washed and incubated with a secondary HRP-conjugated antibody and then exposed to developing solution for 2–20 min until the desired blue colour was achieved. The development reaction was then stopped by addition of a stop solution. The absorbance of each well was then measured at 450 nm with a reference wavelength of 655 nm using a Fluostar Omega^®^ microplate reader (BMG Labtech). To correct the data for cell numbers, the cells were washed and a crystal violet assay performed. All data analysis was performed using BMG Analysis Software (BMG Labtech). The experiment was performed in duplicate on three separate occasions. The percentage of the total STAT3 which was phosphorylated at tyrosine 705 was then calculated in accordance with the manufacturer’s instructions. The percentage data were subjected to angular transformation to parametric data for graphical representation and statistical analysis.

To confirm that changes in STAT-3 phosphorylation state corresponded with modulation of IL-8 expression, simultaneous experiments were performed in which replicate cultures in 96-well plates were exposed to the same conditions as described above. The supernatants were harvested after 4 h and IL-8 release investigated by ELISA.

### Statistical analysis

Statistical analysis was performed using SPSS (IBM, Chicago, USA). The type 1 error alpha for a 2-sided probability was set at 0.05.

Tissue real-time PCR data and real-time PCR data investigating changes in IL-8 mRNA expression in cultured OKF6/TERT-2 cells were analysed by performing Levene’s test on the variance of the natural log-transformed 2^−∆CT^ values followed by an independent *t* test to compare two means (significance was set at *P* < 0.05).

Statistical analysis of ELISA data was performed by log transformation followed by ANOVA with a post hoc Bonferroni correction.

The standardised GeneBLAzer data were subjected to Levene’s test of homoscedasticity. Since the variance of all the data was not significantly different, the statistical difference between mean values for treatments and controls in each experiment was determined by linear ANOVA and a post hoc least significant difference test with Holm−Bonferroni (H–B) correction (significance was set at *P* = <0.05/H–B correction factor).

The angular-transformed NF-κB or STAT-3 data were subjected to Levene’s test of homoscedasticity. Again, since the variance of all the data was not significantly different, the statistical difference between mean values for treatments and controls in each experiment was determined by linear ANOVA and a post hoc least significant difference test with H–B correction (significance was set at *P* = <0.05/H–B correction factor).

## Results

### α7nAChR mRNA expression is elevated in diseased periodontal tissues

**Fig. 1:**
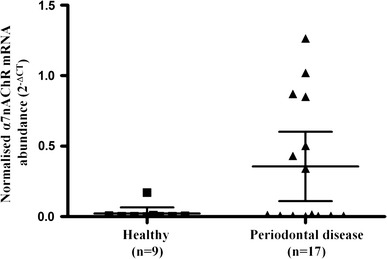
α7nAChR mRNA expression is upregulated in the tissue of patients with periodontal disease. RNA was isolated from tissue samples from healthy patients and those with periodontal disease and analysed for α7nAChR mRNA expression by real-time PCR. α7nAChR mRNA expression was normalised against a housekeeping gene, *RNA Polymerase II*. The data presented represent the mean with 95 % confidence intervals of the normalised α7nAChR mRNA expression for the two cohorts calculated by the 2^−ΔCT^ method. Statistical analysis involved Levene’s test of homoscedasticity on the natural log-transformed 2^−∆CT^ values followed by an independent *t* test to compare two means (*P < 0.05)

The mean level of α7nAChR mRNA expression was elevated in the tissue of 17 patients with periodontal disease when compared with 9 control tissue samples (periodontal disease 2^−ΔCT^ = 0.46 vs healthy 2^−ΔCT^ = 0.24, *P* = 0.017, Fig. 1).

### α7nAChR agonist PHA-543613 HCl inhibits *P. gingivalis*-induced expression of IL-8 by OKF6/TERT-2 cells

**Fig. 2:**
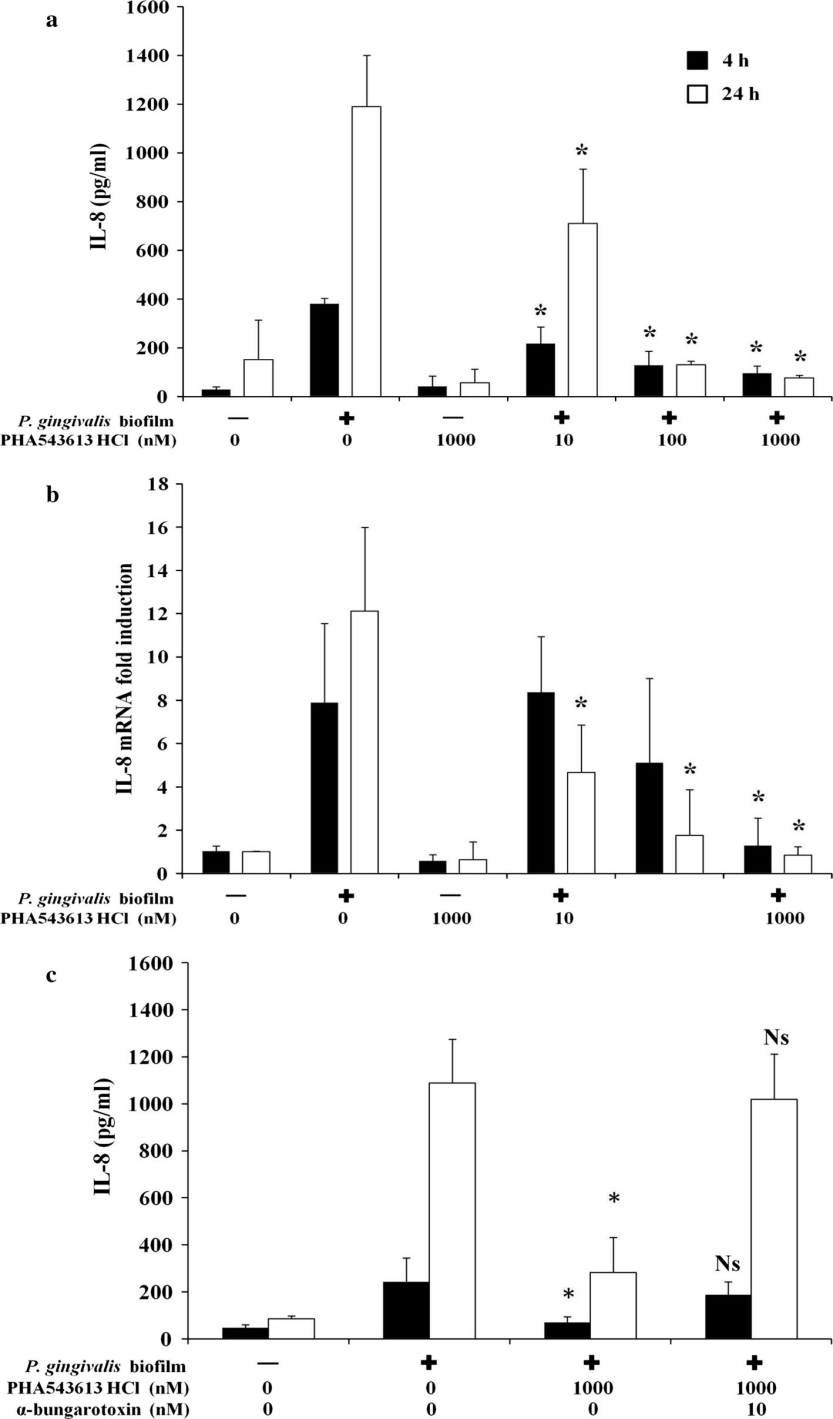
PHA-543613 HCl inhibits *P. gingivalis-*induced expression of IL-8 by OKF6/TERT-2 oral keratinocytes through specific activation of α7nAChR. *P. gingivalis-*induced release of IL-8 by OKF6/TERT-2 oral keratinocytes was inhibited by the α7AChR agonist PHA-543613 HCl (**a**). The inhibition of release by the α7AChR agonist PHA-543613 HCl was mediated at the transcriptional level (**b**). The specific role of α7AChR in mediating the inhibition of *P. gingivalis-*induced expression and release of IL-8 was demonstrated using the specific antagonist, α-bungarotoxin (**c**). IL-8 release into bathing supernatants was determined by ELISA and changes in IL-8 mRNA expression by real-time PCR. ELISA data are presented as the mean IL-8 release with standard deviation (SD) and are derived from duplicate wells of 3 independent experiments (*n* = 3). Statistical analysis of ELISA data was performed by log transforming the data followed by ANOVA with a post hoc Bonferroni corrected *t* test (*P < 0.05). Real-time PCR data are presented as the fold induction change (±SD) in IL-8 mRNA expression in relation to the unstimulated control (2^−ΔΔCT^). The data are derived from duplicate wells of 3 independent experiments (*n* = 3). Statistical analysis of real-time PCR data was performed by Levene’s test of homoscedasticity on the natural log-transformed 2^−∆CT^ values followed by an independent *t* test to compare two means (*P < 0.05)

Stimulation of OKF6/TERT-2 cells with a *P. gingivalis* biofilm for 4 h induced significant release of IL-8 in control unstimulated cells from 28.79 ± 11.68 pg/ml to 380.95 ± 22.03 pg/ml (*P* < 0.001) (Fig. 2a). *P. gingivalis*-induced IL-8 release was inhibited by PHA-543613 HCl (10 nM = 217.01 ± 68.72 pg/ml, *P* = 0.043; 100 nM = 127.71 ± 58.83 pg/ml, *P* = 0.014 and 1,000 nM = 94.98 ± 29.65 pg/ml, *P* = 0.012) (Fig. 2a). Similar effects were seen at 24 h, with *P. gingivalis* inducing significant release of IL-8 in control unstimulated cells from 118.67 ± 61.12 pg/ml to 1,190.67 ± 209.57 pg/ml (*P* = 0.004) (Fig. 2a). Again, this induction of IL-8 release was inhibited by PHA-543613 HCl (10 nM = 710.88 ± 222.67 pg/ml, *P* = 0.046; 100 nM = 129.85 ± 15.69 pg/ml, *P* = 0.013 and 1,000 nM = 76.10 ± 10.92 pg/ml, *P* = 0.011) (Fig. 2a).

The inhibition of IL-8 production was mediated at the transcriptional level (Fig. 2b). IL-8 mRNA expression increased 7.90 ± 3.65-fold after 4 h stimulation with *P. gingivalis* (*P* = 0.032). IL-8 mRNA expression levels were not significantly different when cells were pre-stimulated with 10 and 100 nM PHA-543613 HCl. However, a significant reduction in IL-8 mRNA was observed after 4 h when cells were pre-stimulated with 1,000 nM PHA-543613 HCl (1.28 ± 1.28-fold, *P* = 0.011). At 24 h, *P. gingivalis* increased IL-8 mRNA expression 12.11 ± 3.86-fold (*P* = 0.002). However, in the presence of PHA-543613 HCl, IL-8 mRNA levels were significantly lower with all concentrations investigated (10 nM = 4.66 ± 2.56-fold, *P* = 0.041; 100 nM = 1.76 ± 3.90-fold, *P* = 0.014 and 1,000 nM = 0.84 ± 1.28-fold, *P* = 0.011) (Fig. 2b).

The reduction in IL-8 expression was unrelated to cell viability as determined by a lactate dehydrogenase activity assay (data not shown).

To confirm that the effects of PHA-543613 HCl were specifically mediated by α7nAChR, experiments were repeated using a specific α7nAChR antagonist, α-bungarotoxin. The *P. gingivalis* biofilm induced significant IL-8 release from OKF6/TERT-2 cells at 4 h (control = 47.74 ± 12.27 pg/ml and biofilm = 221.86 ± 50.57 pg/ml; *P* < 0.001) and 24 h (control = 85.84 ± 12.03 pg/ml and biofilm = 1,088.80 ± 185.96 pg/ml; *P* < 0.001). In addition, the *P. gingivalis* biofilm-induced IL-8 release was again inhibited by 1,000 nM PHA-543613 HCl at 4 h (biofilm = 221.86 ± 50.57 pg/ml and biofilm + 1,000 nM PHA-543613 HCl = 35.42 ± 11.59 pg/ml; *P* < 0.05) and 24 h (biofilm = 1,088.80 ± 185.96 pg/ml and biofilm + 1,000 nM PHA-543613 HCl = 70.80 ± 11.59 pg/ml; *P* < 0.05) (Fig. 2c). α-Bungarotoxin alone or in combination with PHA-543613 HCl had no effect on IL-8 release from OKF6/TERT-2 cells (data not shown). In addition, α-bungarotoxin had no effect on *P. gingivalis* biofilm-induced IL-8 release from OKF6/TERT-2 cells (data not shown). However, α-bungarotoxin abolished PHA-543613 HCl-mediated downregulation of *P. gingivalis*-induced IL-8 release from OKF6/TERT-2 cells at 4 h (biofilm = 221.86 ± 50.57 pg/ml and biofilm + 1,000 nM PHA-543613 HCl + 10 nM α-bungarotoxin = 188.19 ± 54.29 pg/ml; *P* > 0.05) and at 24 h (biofilm = 1,088.80 ± 185.96 pg/ml and biofilm + 1,000 nM PHA-543613 HCl + 10 nM α-bungarotoxin = 1,018.33 ± 192.72 pg/ml; *P* > 0.05) (Fig. 2c).

### *P. gingivalis* LPS and PHA-543613 HCl modulate the release of ACh from OKF6/TERT-2 cells

**Fig. 3:**
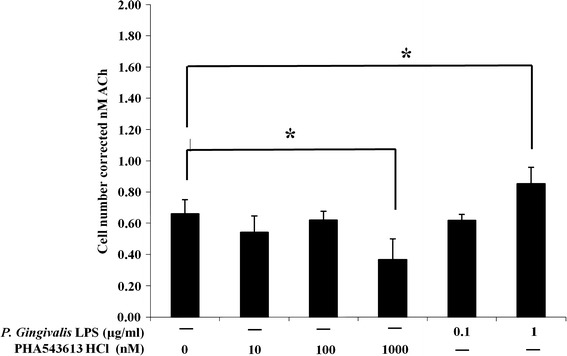
*P. gingivalis* LPS and PHA-543613 HCl modulate the release of ACh from OKF6/TERT-2 cells. The concentration of ACh in cell culture supernatants was determined using the GeneBLAzer M3 CHO-K1-*bla* cell reporter assay. The data represent the total concentration of ACh (with SD) in supernatants after a 10-min incubation period with a correction for cell number. The data are derived from duplicate wells of 3 independent experiments (*n* = 3). The data were standardised and statistical analysis performed by ANOVA and a post hoc Holm−Bonferroni (H–B) corrected least significant difference test (*P < 0.05/H–B)

OKF6/TERT-2 cells were either left unstimulated (basal) or stimulated with *P. gingivalis* LPS or PHA-543613 HCl for 4 h. A 10-min snapshot of ACh release into bathing media was then determined using the GeneBLAzer M3 CHO-K1-*bla* cell reporter assay. The basal release level of ACh from OKF6/TERT-2 cells was 0.66 ± 0.09 nM over a period of 10 min (Fig. 3). No significant change in basal ACh release was observed with 10 and 100 nM PHA-543613 HCl or 0.1 μg/ml *P. gingivalis* LPS. ACh release was significantly downregulated from basal levels in response to 1,000 nM PHA-543613 HCl to 0.37 ± 0.13 nM over a period of 10 min (*P* = 0.031). In contrast, there was a significant increase in ACh release from basal levels by cells stimulated with 1 μg/ml *P. gingivalis* LPS to 0.83 ± 0.91 nM over a period of 10 min (*P* = 0.042) (Fig. 3).

### PHA-543613 HCl regulates the expression of IL-8 in *P. gingivalis*-stimulated OKF6/TERT-2 cells by inhibiting phosphorylation of the NF-κB p65 subunit

**Fig. 4:**
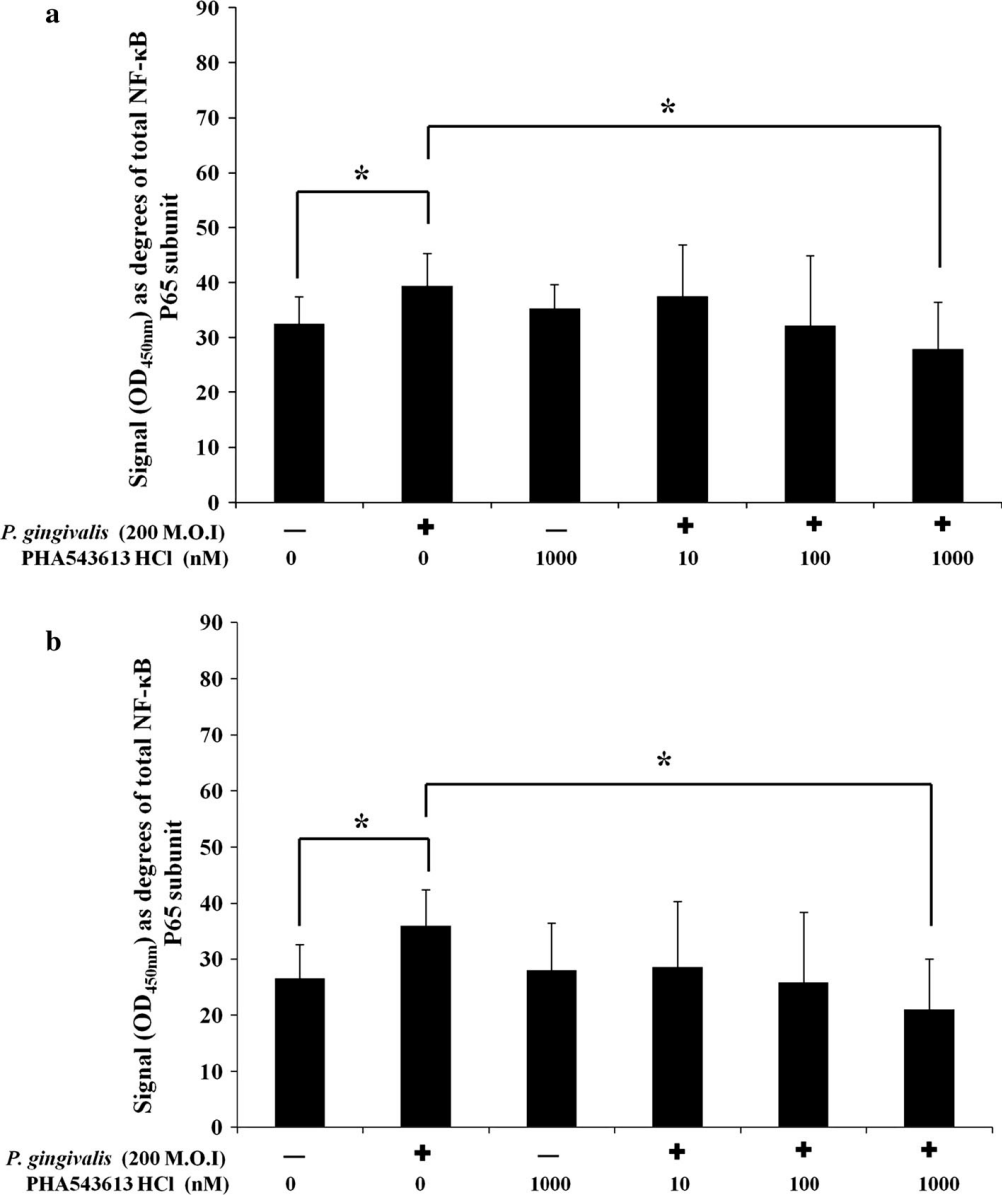
The α7AChR agonist PHA-543613 HCl inhibits *P. gingivalis*-induced phosphorylation of the NF-κB p65 subunit at serine 536 and 468. The percentage of the NF-κB p65 subunit phosphorylated at serine residues 536 (**a**) and 468 (**b**) was calculated. The data represent the mean angular-transformed percentage of NF-κB p65 subunit phosphorylated at each residue with the SD. The data are derived from duplicate wells of 3 independent experiments (*n* = 3). Statistical analysis was performed by ANOVA and a post hoc Holm−Bonferroni (H–B) corrected least significant difference test (*P < 0.05/H–B)

Exposure of OKF6/TERT-2 cells to dead *P. gingivalis* or PHA-543613 HCl for 30 min had no significant effect on total NF-κB p65 subunit levels (data not shown). Stimulation of OKF6/TERT-2 cells with dead *P. gingivalis* caused significantly elevated phosphorylation of the NF-κB p65 subunit at serine 468 from 32.51° ± 4.92° (29 %) in control cells to 39.51 ± 4.92 (40 %) in stimulated cells (*P* = 0.016) (Fig. 4a). In addition, stimulation also caused significantly elevated phosphorylation of the NF-κB p65 subunit at serine 536 from 26.56 ± 6.02 (23 %) in control cells to 36.62° ± 6.38° (31 %) in stimulated cells (*P* = 0.013) (Fig. 4b). There was a significant decrease in *P. gingivalis*-induced NF-κB activation when conducted in the presence of 1,000 nM PHA-543613 HCl. Phosphorylation of the NF-κB p65 subunit at serine 468 was reduced from 39.51 ± 4.92 (40 %) to 27.92 ± 8.52 (23 %) (*P* = 0.011) (Fig. 4a) and serine 536 from 36.62° ± 6.38° (31 %) to 21.04 ± 8.99 (22 %) (*P* = 0.014) (Fig. 4b). Linear ANOVA analysis showed there was a dose-dependent reduction in phosphorylation of serine 468 (*P* < 0.05) and serine 536 (*P* < 0.05) in the presence of PHA-543613 HCl but only the 1,000 nM concentration reached statistical significance in both cases. Analysis of IL-8 release into bathing supernatants from simultaneous cultures showed that the changes in IL-8 release correlated with changes in the phosphorylation state of the NF-κB p65 subunit (data not shown).

### PHA-543613 HCl regulates the expression of IL-8 in *P. gingivalis*-stimulated OKF6/TERT-2 cells by maintaining phosphorylation of STAT-3

**Fig. 5:**
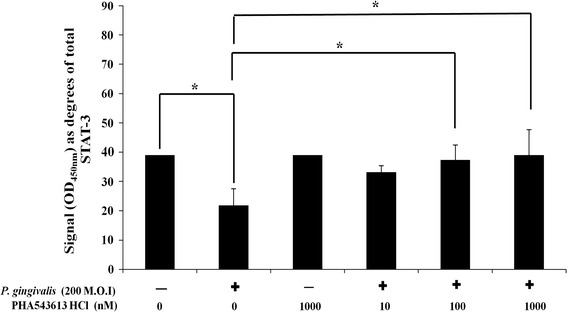
The α7AChR agonist PHA-543613 HCl reverses the *P. gingivalis*-induced decrease in phosphorylation of STAT-3 at tyrosine 705. The percentage of STAT-3 phosphorylated at tyrosine 705 was therefore calculated and angular-transformed to parametric data for graphical representation and statistical analysis. The data represent the mean angular-transformed percentage of STAT-3 phosphorylated at tyrosine 705 with the SD. The data are derived from duplicate wells of 3 independent experiments (*n* = 3). Statistical analysis was performed by ANOVA and a post hoc Holm−Bonferroni (H–B) corrected least significant difference test (*P < 0.05/H–B)

Exposure of OKF6/TERT-2 cells to dead *P. gingivalis* or PHA-543613 HCl had no significant effect on total STAT-3 levels (data not shown). Stimulation of OKF6/TERT-2 cells with dead *P. gingivalis* caused significantly (*P* = 0.022) decreased phosphorylation of STAT-3 at tyrosine 705 from 39.02° ± 0.01° (40 %) in control cells to 21.77° ± 5.74° (14 %) in stimulated cells (*P* = 0.011) (Fig. 5). In comparison to *P. gingivalis*-stimulated cells alone (21.77° ± 5.74°; 14 %), levels of STAT-3 phosphorylated at tyrosine 705 were significantly higher in cells stimulated with *P. gingivalis* in the presence of 100 nM (37.42° ± 5.12°, 38 %; *P* = 0.024) and 1,000 nM (39.07 ± 8.67, 40 %, *P* = 0.013) PHA-543613 HCl (Fig. 5). Linear ANOVA analysis also showed there was a dose-dependent increase in levels of STAT-3 phosphorylated at tyrosine 705 in the presence of PHA-543613 HCl (*P* < 0.05) but only the 100 and 1,000 nM concentrations reached statistical significance. Analysis of IL-8 release into bathing supernatants from simultaneous cultures showed that changes in IL-8 release correlated with changes in the phosphorylation state of STAT-3 (data not shown).

## Discussion

α7nAChR signalling has anti-inflammatory effects on epithelial cells [14–17, 23]. In agreement, this study showed α7nAChR signalling inhibits *P. gingivalis*-induced expression of IL-8 by oral keratinocytes. α7nAChR exerts its anti-inflammatory effects in monocytes through inhibited NF-κB activation [18]. This study suggests that α7nAChR mediates similar effects in epithelial cells. NF-κB exists in cells in an inactivated form and IL-8 gene transcription is reliant on NF-κB activation, which occurs through phosphorylation of the NF-κB p65 subunit and degradation of the inhibitory protein IκB [33, 34]. In this study, α7nAChR activation inhibited *P. gingivalis* LPS-induced phosphorylation of the NF-κB p65 subunit at serine 468 and 536 in oral keratinocytes. Phosphorylation of the NF-κB p65 subunit at serine 536 has been shown to play a role in NF-κB activation [35, 36]. In contrast, phosphorylation at serine 468 has been shown to play an important role in NF-κB ubiquitination and degradation [37]. Despite their opposing functions, phosphorylation at serine 536 and 468 occurs simultaneously [36]. Therefore, NF-κB activation and regulation may go hand in hand. Indeed, phosphorylation of serine 468 is suggested to be a hallmark of constitutively activated NF-κB in chronic inflammatory diseases [38]. Phosphorylation of serine 536 of the NF-κB p65 subunit has been demonstrated to be important in transcription of the IL-8 gene [39]. However, at present, the role that phosphorylation of serine 468 plays in IL-8 transcription is unknown.

Previous studies have demonstrated a role for STAT-3 in suppression of IL-8 transcription [40]. In macrophages, the anti-inflammatory effects of α7nAChR signalling are in part driven by activation of STAT-3 [19]. This study demonstrates that a similar phenomenon occurs in epithelial cells. Indeed, PHA-543613 HCl inhibited *P. gingivalis* LPS-induced expression of IL-8 by oral keratinocytes through maintained STAT-3 tyrosine 705 phosphorylation. Phosphorylation of tyrosine 705 is reported to induce STAT-3 activation [41], which in turn causes dimerisation and binding to target genes [42] as well as promoting suppressor of cytokine signalling 3 (SOCS-3) activation [43, 44]. In macrophages, α7nAChR exerts its anti-inflammatory effects by direct activation of STAT-3 and not through increased SOCS-3 activity [19]. In this study, activation of SOCS-3 was not measured. Therefore, although it can be concluded that direct activation of STAT-3 is responsible for α7nAChR-mediated inhibition of IL-8 expression by oral keratinocytes, it is still unknown whether this occurs through SOCS-3-dependent or -independent mechanisms.

In this study, stimulation of oral keratinocytes with *P. gingivalis* LPS induced a significant decrease in the percentage of STAT-3 phosphorylated at tyrosine 705. This decrease in phosphorylation coincided with increased IL-8 expression (data not shown). In contrast, studies using live *P. gingivalis* have shown it to induce an increase in phosphorylation of STAT-3 tyrosine 705 in gingival epithelial cells [45, 46]. This increased activation of STAT-3 is suggested to be related to the process of internalisation and intracellular adaptation [45]. Therefore, the differential finding in our study may be related to the use of *P. gingivalis* LPS as a stimulator. Indeed, different *P. gingivalis* antigens have in fact been shown to have differential effects on STAT-3 phosphorylation in monocytes. Furthermore, in agreement with the data presented in this study, *P. gingivalis* LPS has been found to downregulate STAT-3 activation [47].

Oral keratinocytes can synthesise and release ACh [21] and evidence suggests that levels are elevated in oral pathologies [3]. The mechanisms controlling release of ACh from non-neuronal cells are at present poorly understood. Placental models have demonstrated a role for organic cation transporter 3 (OCT-3). Pharmacological evidence suggests that oral epithelial cells express OCTs [48]. Furthermore, data from our laboratory suggest oral keratinocytes express OCT-3 mRNA (data not shown). However, at present, the exact mechanisms by which oral keratinocytes mediate the synthesis and release of ACh remain unknown. The data show that *P. gingivalis* LPS can trigger an increase in ACh release from oral keratinocytes. In contrast, agonist-induced α7nAChR signalling triggers a decrease in the release of ACh. Although these findings are in the preliminary stages, it is interesting to speculate that during inflammation the release of ACh from oral keratinocytes is tightly regulated. This regulation may be important in ensuring the immune response is appropriate for the perceived threat. Furthermore, as oral keratinocytes, fibroblasts and numerous immune cells express α7nAChR, a complex network of autocrine and paracrine non-neuronal signalling mechanisms can be hypothesised to be operating within the periodontium that may play a role in periodontal disease pathogenesis. Indeed, this study shows that α7nAChR mRNA levels are elevated in the tissue of patients with periodontal disease. Furthermore, our data suggest that the elevated α7nAChR mRNA expression in diseased periodontal tissue is due to an increased influx of α7nAChR^+^ immune cells (data not shown). The precise α7nAChR^+^ immune cell subsets in diseased periodontal tissue and their role in disease pathogenesis are currently a matter for further investigation.

The results presented here indicate that non-neuronal ACh and α7nAChR may play a role in regulating pathogen-induced immune response at epithelial surfaces. They may also play a role in the pathogenesis of epithelial inflammatory pathologies such as periodontal disease. However, further research is required to investigate the physiological and immunoregulatory functions of non-neuronal ACh/α7nAChR-mediated signalling mechanisms within periodontal tissues.
